# Superior cuproptotic efficacy of diethyldithiocarbamate-Cu_4_O_3_ nanoparticles over diethyldithiocarbamate-Cu_2_O nanoparticles in metastatic hepatocellular carcinoma

**DOI:** 10.3389/fphar.2024.1388038

**Published:** 2024-07-15

**Authors:** Marwa M. Abu-Serie, Assem Barakat, Sherif Ramadan, Noha Hassan Habashy

**Affiliations:** ^1^ Medical Biotechnology Department, Genetic Engineering and Biotechnology Research Institute (GEBRI), City of Scientific Research and Technological Applications (SRTA-City), Alexandria, Egypt; ^2^ Department of Chemistry, College of Science, King Saud University, Riyadh, Saudi Arabia; ^3^ Chemistry Department, Michigan State University, East Lansing, MI, United States; ^4^ Department of Chemistry, Benha University, Benha, Egypt; ^5^ Biochemistry Department, Faculty of Science, Alexandria University, Alexandria, Egypt

**Keywords:** metastatic liver cancer, diethyldithiocarbamate-Cu_4_O_3_ nanocomplex (DC(I+II)NPs), diethyldithiocarbamate-Cu_2_O nanocomplex (DC(I)NPs), stemness genes, cuproptosis induction, oxidant activity

## Abstract

Metastatic hepatocellular carcinoma (HC) is a serious health concern. The stemness of cancer stem cells (CSCs) is a key driver for HC tumorigenesis, apoptotic resistance, and metastasis, and functional mitochondria are critical for its maintenance. Cuproptosis is Cu-dependent non-apoptotic pathway (mitochondrial dysfunction) via inactivating mitochondrial enzymes (pyruvate dehydrogenase “PDH” and succinate dehydrogenase “SDH”). To effectively treat metastatic HC, it is necessary to induce selective cuproptosis (for halting cancer stemness genes) with selective oxidative imbalance (for increasing cell susceptibility to cuproptosis and inducing non-CSCs death). Herein, two types of Cu oxide nanoparticles (Cu_4_O_3_ “C(I + II)” NPs and Cu_2_O “C(I)” NPs) were used in combination with diethyldithiocarbamate (DD, an aldehyde dehydrogenase “ALDH” inhibitor) for comparative anti-HC investigation. DC(I + II) NPs exhibited higher cytotoxicity, mitochondrial membrane potential, and anti-migration impact than DC(I) NPs in the treated human HC cells (HepG2 and/or Huh7). Moreover, DC(I + II) NPs were more effective than DC(I) NPs in the treatment of HC mouse groups. This was mediated via higher selective accumulation of DC(I + II) NPs in only tumor tissues and oxidant activity, causing stronger selective inhibition of mitochondrial enzymes (PDH, SDH, and ALDH2) than DC(I)NPs. This effect resulted in more suppression of tumor and metastasis markers as well as stemness gene expressions in DC(I + II) NPs-treated HC mice. In addition, both nanocomplexes normalized liver function and hematological parameters. The computational analysis found that DC(I + II) showed higher binding affinity to most of the tested enzymes. Accordingly, DC(I + II) NPs represent a highly effective therapeutic formulation compared to DC(I) NPs for metastatic HC.

## 1 Introduction

Hepatocellular carcinoma (HC) is one of the most common types of primary malignant liver tumors and the second-leading cause of cancer death (Liu and Qian, 2021). Its incidence and mortality rates are increasing, making it a challenging global health issue. According to estimations, over a million people will be diagnosed with HC annually by 2025 (Philips et al., 2021; Wang et al., 2021). Metastasis is the main cause of HC death; the lungs are the most frequent site of metastatic HC (Terada and Maruo, 2013; Chang et al., 2019). The stemness of liver cancer stem cells (CSCs) represents the main driver of HC initiation, progression, therapeutic resistance, recurrence, and metastasis (Chang et al., 2019; Jing et al., 2021). CSCs maintain stemness by up-regulating unique genes, including CD133 (prominin1) and aldehyde dehydrogenase (ALDH)2, to activate Notch and Wnt/β-catenin signaling pathways, resulting in the expression of self-renewal transcription factors (SOX2, OCT-4, and NANOG) and drug efflux transporters (e.g., ATP binding cassette subfamily G member 2, ABCG2). This condition confers more metastatic and chemoresistance effects (Wang and Sun, 2018; Liu and Qian, 2021; Zhang and Fu, 2021). The latter is also mediated by glutathione-S-transferase (GST), particularly GST π (GSTP1), which is highly expressed in malignant tumors and maintains stemness by detoxifying reactive oxygen species (ROS) and DNA-damaging compounds, conferring apoptosis resistance (Cui et al., 2020; Niitsu et al., 2022). The stemness markers in HC are also associated with overexpression of the fetal liver cell marker (α-fetoprotein, AFP), which is an indicator of poorly differentiated HC (Sell, 2008). Functional mitochondria (tricarboxylic acid cycle (TCA) and respiratory chain) are essential for maintaining the stemness of apoptotic resistance CSCs (Loureiro et al., 2017; Yadav et al., 2020). Therefore, finding other forms of regulated cell death (apoptosis) depending on mitochondrial dysfunction is critical for effectively eradicating metastatic seeds, CSCs.

Cuproptosis, which is based on cellular Cu accumulation, mediates nonapoptotic mitochondrial cell death via inactivation of mitochondrial lipoylated TCA enzymes (e.g., pyruvate dehydrogenase complex (PDH), α-ketoglutarate dehydrogenase, 2-oxoadipate dehydrogenase, and branched-chain ketoacid dehydrogenase) and mitochondrial Fe-S cluster-containing enzymes (Lv et al., 2022). This high intracellular Cu binds specifically to the lipoyl moiety of dihydrolipoamide S-acetyltransferase (DLAT, regenerating lipoamide cofactor), a component E2 of PDH, which is a crucial enzyme for the transformation of glycolysis to TCA. This binding results in disulfide-mediated aggregation of Cu-lipoylated proteins and then halts TCA (Tang et al., 2022). On the other hand, this accumulated Cu binds to sulfur and displaces the catalytic iron atom of mitochondrial Fe-S cluster proteins, causing cluster degradation (Vallieres et al., 2017; Tang et al., 2022). Mitochondrial Fe-S cluster proteins are key cofactors for electron transfer complexes of the respiratory chain with dual functions in TCA (e.g., succinate dehydrogenase “SDH”, complex II”), whereas nuclear Fe-S cofactors are involved in DNA replication, DNA repair, and genome integrity (regulator of telomere length helicase 1, RTEL1) (Paul and Lill, 2015; Read et al., 2021; Li et al., 2022). The latter contributes to stability of the whole genome and telomere and is associated with HC risk (Margalef et al., 2018). The resultant toxic lipoylated protein aggregation and mitochondrial Fe-S protein degradation caused by Cu accumulation induce mitochondrial proteotoxic stress, causing loss of mitochondrial membrane potential (MP) and ultimately cell death, particularly in the case of proteasomal dysfunction (Li et al., 2022; Tang et al., 2022). Notably, the depletion of glutathione (GSH, a nonenzymatic antioxidant) makes cancer cells more susceptible to cuproptosis (Tsvetkov et al., 2022).

As a consequence, in this study, green chemically synthesized Cu oxide nanoparticles (NPs) were used to improve cuproptotic selectivity in combination with diethyldithiocarbamate (DD). The latter (an active metabolite of FDA-approved anti-alcoholism remedy) was used, herein as Cu oxide NPs ionophore to maximize the cuproptosis effect via its potency for suppressing ALDH, depleting GSH, and inhibiting the S26 proteasome (a major protease for degrading damaged toxic proteins) (Cvek et al., 2008; Abu-Serie and Eltarahony, 2021; Abu-Serie and Abdelfattah, 2023; Abu-Serie et al., 2024; Abu-Serie, 2024). Abu-Serie and Eltarahony (2021) illustrated that a new nanocomplex of cuprous oxide NPs and DD (DC(I) NPs) can repress the growth of human lung, colon, liver, and prostate cancer cells at IC_50_ < 2.5 μg/mL by inhibiting ALDH1A1 and elevating the cellular content of ROS. Furthermore, this nanocomplex (DC(I) NPs) exhibited potent anti-migration efficacy on the above-mentioned cell lines (Abu-Serie and Eltarahony, 2021). Another recent study by the author demonstrated that a unique nanocomplex of DD-Cu_4_O_3_ NPs (DC(I + II) NPs) can eradicate metastatic breast cancer by suppressing CSC genes and the metastasis marker (matrix metalloproteinase (MMP)9), as well as disturbing redox markers, using MDA-MB 231 cells and an orthotopic animal model (Abu-Serie and Abdelfattah, 2023). These recent findings prompted us to investigate the cuproptosis and oxidative alteration effects of DC(I + II) NPs in comparison with DC(I) NPs for suppressing the HC markers (α-fetoprotein and GST), stemness and GSTP1 genes, ALDH2 activity, and metastasis markers (MMP9 gene and TWIST1) in the treatment of metastatic HC. The current study also used molecular docking analysis to predict the inhibitory mechanisms of these nanocomplexes on the activity of the biochemically investigated enzymes, including GSTP1, MMP9, PDH, SDH, and ALDH2.

## 2 Materials and methods

### 2.1 Materials

Copper salts, (3-(4,5-dimethylthiazol-2-yl)-2,5-diphenyltetrazolium bromide (MTT), ethidium bromide, acridine orange, phenobarbital (PB), p-dimethylaminobenzene (DAB), Tris-HCL, glutathione (GSH), and 5,5′-dithiobis2-nitrobenzoic acid (Ellman’s reagent) were purchased from Sigma-Aldrich (Saint Louis, MO, USA). In addition, reagents of mitochondrial enzyme activity assays were from Sigma-Aldrich (Saint Louis, MO, USA). Chitosan and DD were obtained from Acros Organics (Morris Plains, NJ, USA). RPMI 1640 medium, HEPES buffer, phosphate buffer saline (PBS), and fetal bovine serum (FBS) were supplied from GIBCO (Grand Island, NY, USA). Primary antibodies to Ki-67 (Cat#PA1-21520) and Twist family bHLH transcription factor 1 (TWIST1, Cat #PA5-116628), tetramethylrhodamine ethyl ester (TMRE) dye, GeneJET RNA purification kit, one-step qPCR SYBR green kit, and primers were purchased from Thermo Fisher Scientific (Waltham, MA, USA). α-Fetoprotein (AFP) electrochemiluminescence kit (code# 10121) and liver function kits were obtained from Roche Diagnostics (USA) and Spectrum Diagnostics (Cairo, Egypt), respectively. Other chemicals were analytical grades (El-Nasr Pharmaceutical Chemicals Company, Cairo, Egypt).

### 2.2 Methods

#### 2.2.1 Preparation and characterization of nanocomplexes of DD-Cu oxide NPs

As described in author recent studies, Cu_4_O_3_ NPs and Cu_2_O NPs were green synthesized using copper chloride and copper nitrate as Cu precursors, respectively, in the presence of chitosan and vitamin C. Both copper oxide NPs were well characterized, as mentioned in author’s recent studies, using a zetasizer, X-ray diffractometer (XRD), energy dispersive X-ray analysis (EDX), and electron microscopes. These NPs were mixed with DD (10:1), forming DC(I + II) NPs and DC(I) NPs, respectively. The size and zeta potential of two generated nanocomplexes were assessed and mentioned in the author’s recent studies (Abu-Serie and Eltarahony, 2021; Abu-Serie and Abdelfattah, 2023). Additionally, the scanning electron microscope (SEM, JEOL “JSM-5300”, Japan) was used to demonstrate the morphology of these nanoformulations. The elemental composition of DC(I + II) NPs and DC(I) NPs was identified through energy dispersive X-ray analysis (EDX, JEOL “JEM-1230”, Japan) at a voltage of 200 KV. Concurrently, typical complexes of DD-Cu chloride (DCC) and DD-Cu nitrate (DCN) were prepared in the same ratio.

Importantly, the chemical stability of DC(I + II) NPs and DC(I) NPs was investigated by incubation in conditions (PBS-contained 50% FBS, pH 7.4, 37°C) resembling the biological system by measuring their size and polydispersity index (PDI, aggregation index) throughout 80 h, using a particle size analyzer (Malvern Panalytical, United Kingdom).

#### 2.2.2 *In vitro* assessment of the anti-liver cancer efficacy of the prepared nanoformulations

##### 2.2.2.1 MTT cytotoxicity assay and detection of morphological alteration using the fluorescence microscope

This assay determines the cellular growth inhibition potential based on the intact membrane and active mitochondria. The latter reduces tetrazolium dye (MTT) to insoluble formazan by its oxidoreductase enzymes. Briefly, WRL68 (normal human liver cell line, ATCC CL-48, passage no. “P#”17), HepG2 (human liver cancer cells, ATCC HB-8065, P#35) and Huh7 (PTA-4583, ATCC, P#29) were cultured in EMEM, DMEM, and RPMI 1640, respectively, containing 10% FBS. These cell lines were seeded as 6 × 10^3^ cells/well in 96-well plates and allow to attach for 24 h. Then serial concentrations of nanocomplexes (DC(I + II) NPs and DC(I) NPs), traditional complexes (DCC and DCN), DD, and Cu oxide NPs, as well as their corresponding Cu salts, were added. After 72 h in 5% CO_2_ incubator, MTT was added, incubated for 4 h, discarded, and DMSO was added (Mosmann, 1983). The cell viability was determined by measuring the absorbance of wells at 590 nm (spectrophotometer plate reader, BMG LabTech, Germany). The dose of 50% cell growth inhibition (IC_50_) was estimated using GraphPad Prism version 9, and the morphological variations in the most active compounds-treated cells were recorded using a digital camera-supplemented phase contrast inverted microscope (Olympus, Japan).

More importantly, alteration in the nuclear morphology of the treated cells was recorded using a digital camera-supplemented fluorescence microscope (Olympus, Japan) after 15 min of incubation with dual fluorescence dye (acridine orange-ethidium bromide at a final concentration of 50 μg/mL) and washing with PBS.

##### 2.2.2.2 Flow cytometry analysis of mitochondrial damage

The mitochondrial dysfunction was measured in terms of decreasing MP after staining with cationic dye (TMRE), which accumulates only in active mitochondria and emits red fluorescence that was quantified using flow cytometry (Crowley et al., 2016). After 72 h incubation with the lowest IC_50_ ∼2.7 μg/mL, HepG2 cells were stained with 150 nM TMRE in 25 mM HEPES buffer (pH 7.4) containing 75 mM KCl, 80 mM NaCl, and 25 mM D-glucose. Following 10 min of incubation at 37°C, cells were washed and analyzed by flow cytometry (Partec, Germany) at the FL-3 channel (excitation at 549 nm and emission at 575 nm) with FloMax software for gating cells with low fluorescence relative to the untreated cells.

##### 2.2.2.3 Wound healing (migration) assay

Briefly, 90%-confluent HepG2 cells were scratched and incubated with safe doses (0.1 μg/mL) of nanocomplexes or corresponding typical complexes. The scratched area was pictured at 0 h and 24 h, then measured using ImageJ software to estimate the percentage of migration inhibition.

#### 2.2.3 *In vivo* assessment of anti-metastatic liver cancer potential

##### 2.2.3.1 Experimental design

Swiss albino male mice (n = 75, 20–25 g) were divided into two main groups: the normal healthy group (N, n = 36 mice) and the hepatocellular carcinoma group (HC, n = 39 mice). The animals in the latter group were fed 165 mg DAB/kg body weight (b.wt) and given orally 0.05% PB (1.2 mg/kg b. wt) daily for 6 weeks to induce HC (Pathak and Khuda-Bukhsh, 2007). The metastatic HC was confirmed by hematoxylin and eosin staining of the liver and lung tissues of three mice (Figure 4E). Then the two groups (N and HC) were randomly subdivided into three sub-groups (12 mice each), including untreated, DC(I + II) NPs, and DC(I) NPs. The latter two groups of each main group (i.e., N-DC(I + II) NPs, N-DC(I) NPs, HC-DC(I + II) NPs, and HC-DC(I) NPs) were intraperitoneally injected, three times/week, with 2 mg nanocomplex/kg b. wt for 3 weeks. The doses, route and frequencies of nanocomplexes administration were selected based on a previous study (Abu-Serie, 2023). Throughout the 3 weeks of period treatment, the mice’s body weights were measured every 3 days. On the 8^th^ week, six mice from each group were decapitated by anaesthetization with isoflurane (2%–3%, inhalation), then liver and lung tissues were collected for assessment of the redox parameters (ROS, GSH, lipid peroxidation, and ALDH2). At the termination of the experiment (after 9 weeks), all mice were sacrificed, and then blood and tissues (liver, lung, and spleen) were collected. Blood samples were harvested in EDTA tubes for hematological and AFP assays. Liver, lung, and spleen were weighted relative to the recorded b. wt. Minor portions of tissues were 10% formalin-fixed for histological and immunohistochemical examinations, whereas the remaining portions were stored at −80°C for biochemical and molecular studies.

##### 2.2.3.2 Quantification of AFP level and GST activity, and histological, immunohistochemical, and molecular assessments of tumor tissues

The blood level of AFP (primary liver tumor marker) was determined according to the instructions of AFP chemiluminescent immunoassay kit.

For determination of GST activity, liver nodules of the untreated HC group and liver tissues of other groups were homogenized in PBS and centrifuged (10,000 xg for 30 min) at 4°C. The resulting supernatants were added to 0.1 M PBS (pH 6.5), 1 mM 1-chloro-2,4-dinitrobenzene, and 1 mM GSH. The absorbance of the formed conjugate of CDNB-GSH was measured every minute for 5 min at 340 nm (Habig et al., 1974). The activity was calculated in relation to protein content that was assessed by the Bradford method (Bradford, 1976).

Regarding histological (H&E staining of liver, lung, and spleen) and immunohistochemical (Ki-67 immunostaining of liver and lung tumor tissues as well as TWIST1 immunostaining of liver) investigations, tissue slides were prepared using typical protocols. The obtained immunostaining images were analyzed using CellSens and ImageJ software.

Total RNA was extracted from the tumor tissues (liver and lung) of the untreated and treated HC groups based on the kit’s usual protocol. After RNA quantification, one-step SYBR green master mix qPCR kit with specific primers (Supplementary Table S1) was used to determine the alteration in gene expressions in the untreated HC group (positive control) and treated HC groups relative to the untreated N group (negative control). These genes included ABCG2, prominin 1 (CD133), NOTCH1, WNT1, SOX2, OCT-4, NANOG, GSTP1, telomerase reverse transcriptase (TERT), MMP9, vascular endothelial growth factor (VEGF)A, and cyclin D genes by the equation of 2^−ΔΔCt^.

##### 2.2.3.3 Biodistribution and histological investigation of nanocomplexes’ safety

In a separate experiment, fifteen HC-bearing mice (∼20 g) were divided into three groups (untreated, HC-DC(I + II) NPs, and HC-DC(I) NPs). The two latter groups received intraperitoneal injections of the corresponding nanocomplex, as described above. Mice were then sacrificed, and tissues (liver, lung, spleen, brain, heart, and kidney) were collected for quantification of Cu, which correlates with the accumulated tissue uptake of nanocomplexes using graphite atomic absorption spectroscopy (Analytik Jena AG, Germany).

Moreover, the nanocomplexes’ safety was investigated by H&E staining of various tissues (liver, lung, spleen, brain, and kidney) in the treated N groups *versus* the healthy N group.

##### 2.2.3.4 Evaluation of the selective inactivation impact of nanocomplexes on mitochondrial metabolic enzymes

To investigate the efficacy of nanocomplexes in inducing selective cuproptosis, PDH and SDH inhibition percentages were assessed in liver and lung tissues in all HC subgroups compared to their corresponding N groups. A mitochondrial pellet was prepared by homogenizing tissue in a solution of 10 mM Tris-HCl (pH 7.2), 250 mM sucrose, and 1 mM EDTA. The homogenate was centrifuged (600 xg, 10 min), and the obtained supernatant was further centrifuged at 10,000 xg for 10 min (Munujos et al., 1993; Schwab et al., 2005). After freezing (>15 min) and thawing the resulting pellet, it was suspended in 20 mM Tris-HCl (pH 7.5), 1 mM CaCl_2_, 50 mM KCl, 5 mM MgCl_2_, 1 mL/L Triton X-100, and 250 mM sucrose for PDH activity determination (Schwab et al., 2005). Meanwhile, for the SDH activity assay, this pellet was suspended in 10 mM HEPES, 5 mM potassium phosphate buffer (PPB, pH 7.2), 220 mM sucrose, and 20 mM KCl (Munujos et al., 1993). The activity of PDH was measured in the presence of 0.6 mM INT, phenazine methosulfate in PPB (pH 7.5), and 5 mM pyruvate using the Schwab et al. method [31]. While the SDH activity was assayed following the method of Munujos et al., using 2 mM INT, 20 mM succinate, and 1.2% Cremophor EL (pH 7.4). The percent inhibition of both enzymes was detected by measuring the decrease in color development of the produced formazan at 500 nm compared to the untreated group (Munujos et al., 1993). The protein content was determined according to the Bradford method (Bradford, 1976).

##### 2.2.3.5 Biochemical assessment of redox parameters

To detect the prooxidant selectivity of the tested nanocomplexes, levels of ROS (Socci et al., 1999), GSH (Sedlak and Lindsay, 1968), and lipid peroxidation (Ohkawa et al., 1979), as well as ALDH2 activity (Josan et al., 2013) were determined in the liver and lung tumor tissues of untreated and treated HC-bearing mice compared to N groups. All these indicators were calculated using the corresponding standard curves and in relative to tissue protein content that was quantified using the Bradford method (Bradford, 1976).

##### 2.2.3.6 Investigation of liver function and hematological parameters

The main liver function parameters (alanine aminotransferase (ALT), aspartate aminotransferase (AST), and albumin) were detected in liver homogenate (1 g of liver homogenized in 150 mM Tris-KCl buffer, pH 7.4) (Gaskill et al., 2005). These parameters were measured using commercial colorimetric kits. Additionally, a complete blood count (CBC) was assessed in all groups using the hematology analyzer (Mindray, China).

#### 2.2.4 *In silico* studies

The current study performed a set of computational analyses to predict the complex structures of DD with Cu_2_O [DC(I)] or Cu_4_O_3_ [DC(I + II)] and the inhibitory effects of these complexes on certain enzymes, including GSTP1, PDH (DLAT), SDH, ALDH, and MMP9.

##### 2.2.4.1 2D and 3D structures of the studied compounds and proteins

ChemDraw v.16.0 was used to draw the 2D structures (.mol) of DC(I + II) and DC(I) complexes. The structures were then converted to 3D format (.pdb) using the ChemAxon Chemical Sketch Tool (https://www.rcsb.org/chemical-sketch). The 3D structures of GSTP1 (PDB: 3GUS), DLAT (PDB: 1FYC), SDH (PDB: 1NEN), ALDH2 (PDB: 4FR8), and MMP9 (PDB: 1L6J) were retrieved from the Protein Data Bank (PDB, https://www.rcsb.org/).

##### 2.2.4.2 Docking analysis

The Discovery Studio 2020 Client program (v20.1.0.19295) was used to pre-process the protein structure before further analysis by removing water and ligands. Then the 3D structure of the DC(I + II) or DC(I) complex was docked with GSTP1, DLAT, SDH, ALDH, and MMP9 using the HDOCK server (http://hdock.phys.hust.edu.cn/) (Yan et al., 2017). The highest-scoring docked complexes were used, and their interfaces were visualized and analyzed by the Discovery Studio program.

To study the effect of DC(I + II) and DC(I) on DLAT aggregation, the 3D structure of DLAT or the docked complex of DLAT-Cu complex structures were docked sequentially with another two DLAT structures. The binding affinity between DLAT molecules in the resulting docked complexes was determined and compared. While the iron displacement capability of the studied Cu complexes was evaluated by docking each of these complexes with SDH 3D structure without Fe-S centers. The resulting Cu complex-SDH docked complexes were then docked with Fe-S to examine the binding affinity between SDH and Fe-S in the presence and absence of the Cu complexes. Furthermore, the binding affinity between ALDH or MMP9 and DC(I + II) or DC(I) was evaluated and compared for each enzyme.

##### 2.2.4.3 Binding affinity analysis in the docked complexes

The gained solvation-free energy (change in Gibbs free energy, ΔG) during the interface fomation in the obtained docked complexes was determined using the PDBePISA (Proteins, Interfaces, Structures, and Assemblies) platform (https://www.ebi.ac.uk/msd-srv/prot_int/cgi-bin/piserver) (Krissinel and Henrick, 2007). This server is a key protein interaction analysis tool in proteomics databases and servers. It is commonly utilized to investigate macromolecular interfaces in protein-protein and protein-ligand docked complexes to obtain important information about protein interactions from complex structures. The obtained ΔG value is implemented in the prediction of the binding affinity of protein-protein or protein-ligand docked complexes.

##### 2.2.4.4 Prediction of the competitive inhibitory impacts of DC(I + II) and DC(I) on the studied enzymes

The competitive inhibitory effect of the studied complexes on the target enzymes was analyzed by comparing the amino acid residues in the active site of these enzymes with those in the interfaces of the docked complexes using the Discovery Studio software. The PDBsum web-based database (http://www.ebi.ac.uk/pdbsum) was used to identify the active site residues of the enzymes investigated using their PDB ID (Laskowski et al., 2018).

#### 2.2.5 Statistical analysis

Data is presented as mean ± standard deviation (SD) and replicates derived from at least three independent experiments (n ≥ 3). Data was analyzed with GraphPad Prism version 9.3.1. via one-way analysis of variance (ANOVA), multiple comparisons (Dunnett test), and unpaired *t*-test. Statistical significance was deemed at *p* < 0.05*, <0.005**, and <0.0001***.

## 3 Results

### 3.1 Characterization of the nanocomplexes

**FIGURE 1:**
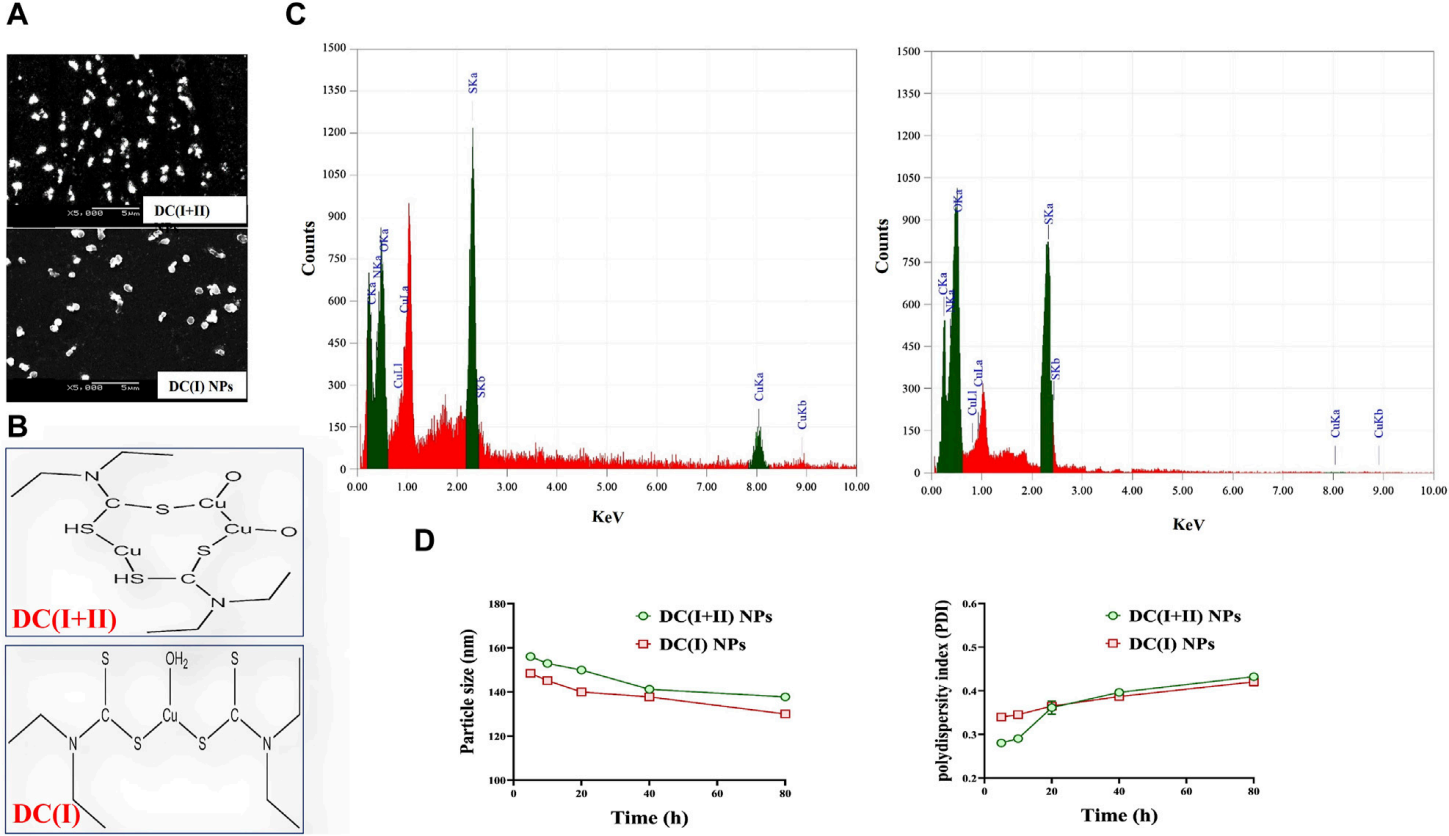
Morphology, ChemDraw 2D structures, elemental composition analysis, and stability of nanocomplexes. **(A)** Scanning electron microscope images of diethyldithiocarbamate (DD)-Cu_4_O_3_ “C(I + II)” nanoparticles (DC(I + II) NPs) and DD-Cu_2_O NPs “C(I)” NPs (DC(I) NPs) (magnification ×5000). **(B)** 2D structures of nanocomplexes of DC(I + II) NPs and DC(I) NPs, as generated by ChemDraw software. **(C)** Elemental composition of nanocomplexes of DC(I + II) NPs and DC(I) NPs, as analyzed by energy dispersive X-ray spectroscopy. **(D)** Biostability of the prepared nanocomplexes during 80 h incubation in phosphate buffer saline-contained 50% fetal bovine serum at 37°C in terms of variations in particle size and aggregation index (PDI). Data are shown as mean ± SD (n = 3).

Herein, DD was nanoformulated by chelating with Cu_4_O_3_ NPs or Cu_2_O NPs, forming semi-spherical-shaped nanocomplexes of DC(I + II) or DC(I), respectively (Figure 1A). As demonstrated in author’s recent studies (Abu-Serie and Eltarahony, 2021; Abu-Serie and Abdelfattah, 2023), these nanocomplexes have mean sizes of 156.5 nm and 148.1 nm, respectively, and their mean zeta potentials were - 4.65 mv and - 20.2 mv, respectively, with mean PDI values of 0.274 and 0.337, respectively. It is worth mentioning that the ChemDraw 2D structures of the studied nanocomplexes (Figure 1B) align with the previously established coordination complexes of DD-Cu (I + II) and DD-Cu (I) (Han et al., 2013). Figure 1D declares the elemental composition including C (19.36% and 17.99%), N (9.00% and 7.71%), O (12.36% and 13.93%), S (22.75% and 15.05%), and Cu (36.52% and 21.39%) for DC(I + II) and DC(I), respectively. Moreover, as illustrated in Figure 1D, there was no discernible shift in the ranges of the nanosizes and PDI values (<0.44) of both nanocomplexes during 80 h incubation in PBS/50%FBS, indicating their stability and acceptable particle distribution without aggregation (homogeneity) in serum condition.

### 3.2 *In vitro* anti-liver cancer efficacy of the prepared nanocomplexes

**FIGURE 2:**
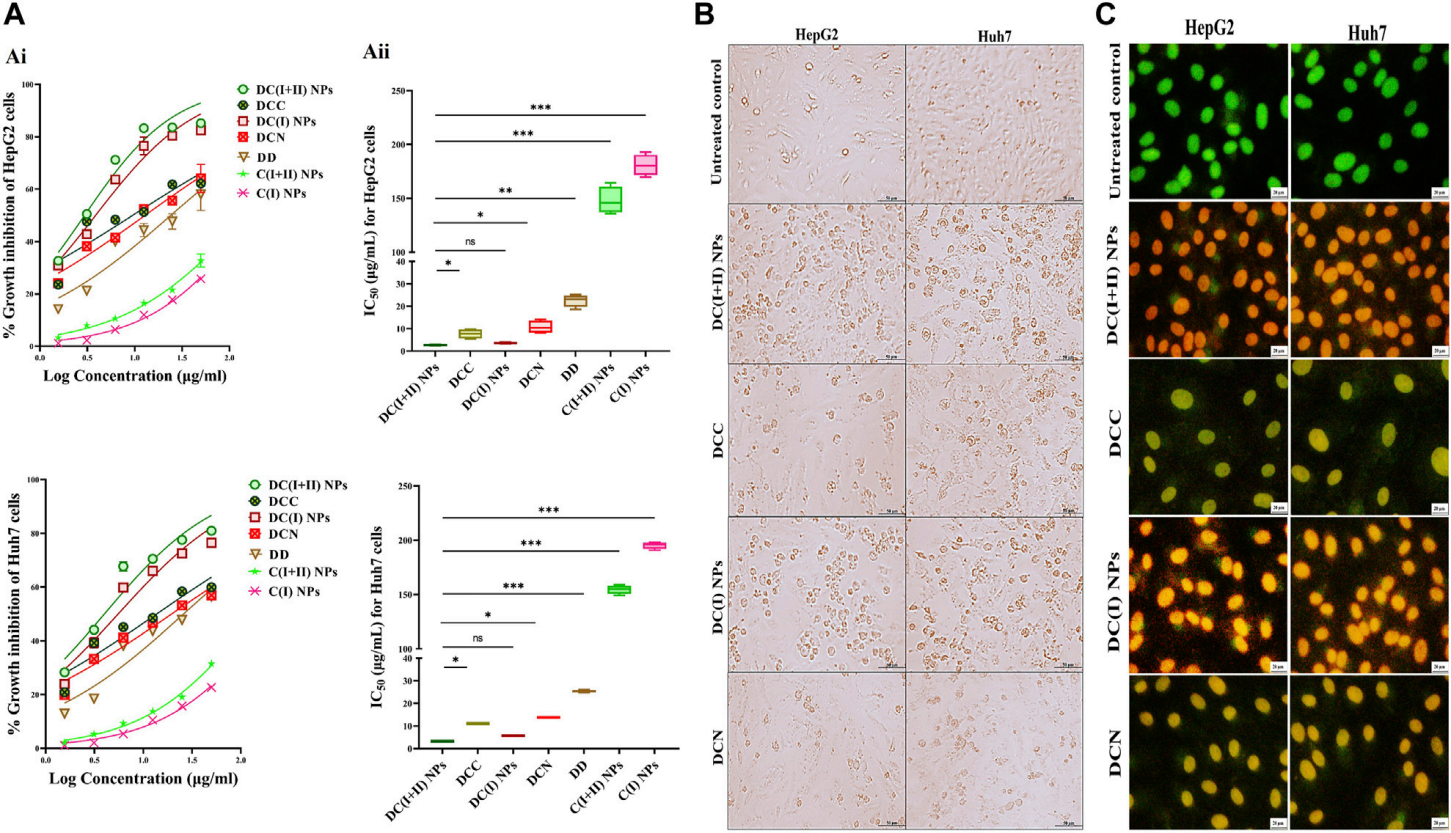
Cytotoxicity on human liver cancer (HepG2 and Huh7) cell lines and cellular morphology alterations using phase contrast and fluorescence microscopes. **(A)** Cytotoxicity on HepG2 and Huh7 using MTT assay as demonstrated by (Ai) dose-response curves and (Aii) IC_50_ values of nanocomplex of diethyldithiocarbamate (DD)-Cu_4_O_3_ “C(I + II)” NPs (DC(I + II) NPs), nanocomplex of DD-Cu_2_O NPs “C(I)” NPs (DC(I) NPs), their corresponding typical complexes (DCC and DCN) of DD with precursors of copper oxide NPs (copper chloride and copper nitrate, respectively), DD, and Cu oxide NPs of C(I + II) NPs and C(I) NPs. Morphological changes of nanocomplexes-treated HepG2 and Huh7 cells, compared to DCC- and DCN-treated cells **(B)** using phase contrast microscope (magnification ×100) and **(C)** after staining with dual nuclear dyes (ethidium bromide and acridine orange), dead cells show yellowish green or yellow and orange or reddish orange nuclei at the early and late cell death, respectively, in comparison to viable green fluorescence nuclei, as demonstrated using fluorescence microscope (magnification ×200). Data are shown as mean ± SD (n = 3). DC(I + II) NPs were compared to other tested compounds, and values are considered statistically significant at *p* < 0.05*, <0.005**, and <0.0001***.

The estimated IC_50_ for normal liver cells demonstrated that nanocomplexes (DC(I + II) NPs and DC(I) NPs) and Cu oxide NPs (C(I + II) NPs and C(I) NPs) had higher values (97.8, 94.5, 99.6, and 97.3 μg/mL, respectively) than traditional complexes (DCC and DCN) and DD (31.1, 29.2, and 14.7 μg/mL, respectively). In a dose-dependent manner (Figure 2Ai), both nanocomplexes exhibited the lowest IC_50_ values (<5 μg/mL) compared to their corresponding typical complexes (<14 μg/mL), DD (≤25 μg/mL), and Cu oxide NPs (>147 μg/mL) for inhibiting HepG2 and Huh7 growth (Figure 2Aii). In terms of IC_50_ values, DC(I + II) NPs (2.71 and 3.26 μg/mL) showed comparable values to DC(I) NPs (4.73 and 3.56 μg/mL) against HepG2 and Huh7 cells, respectively. Additionally, the most severe collapse in morphology of both liver cancer cell lines was observed in DC(I + II) NPs-treated cells, followed by DC(I) NPs-treated cells, compared to other treated cells (Figure 2B). Furthermore, dual nuclear fluorescence dyes were used to discriminate the grades of cell death in the treated HC cell lines. It is based on the acridine orange staining cells with green fluorescence, while ethidium bromide is only taken up by the damaged cells; as damage increases more ethidium bromide is entered, making their nuclei appear yellow to red according to damage degree. This fluorescence staining of these treated cells supported the highest lethal effect of DC(I + II) NPs, as demonstrated by organ fluorescence nuclei in comparison with the yellowish orange nuclei of DC(I + II) NPs, the yellow or yellowish green nuclei of traditional complexes, and the green nuclei of the untreated control (Figure 2C). Because there was no statistically significant difference between HepG2 and Huh7 in the cytotoxicity efficacy of the tested nanocomplexes, the following parameters were only investigated *in vitro* using 1 cell line.

**FIGURE 3:**
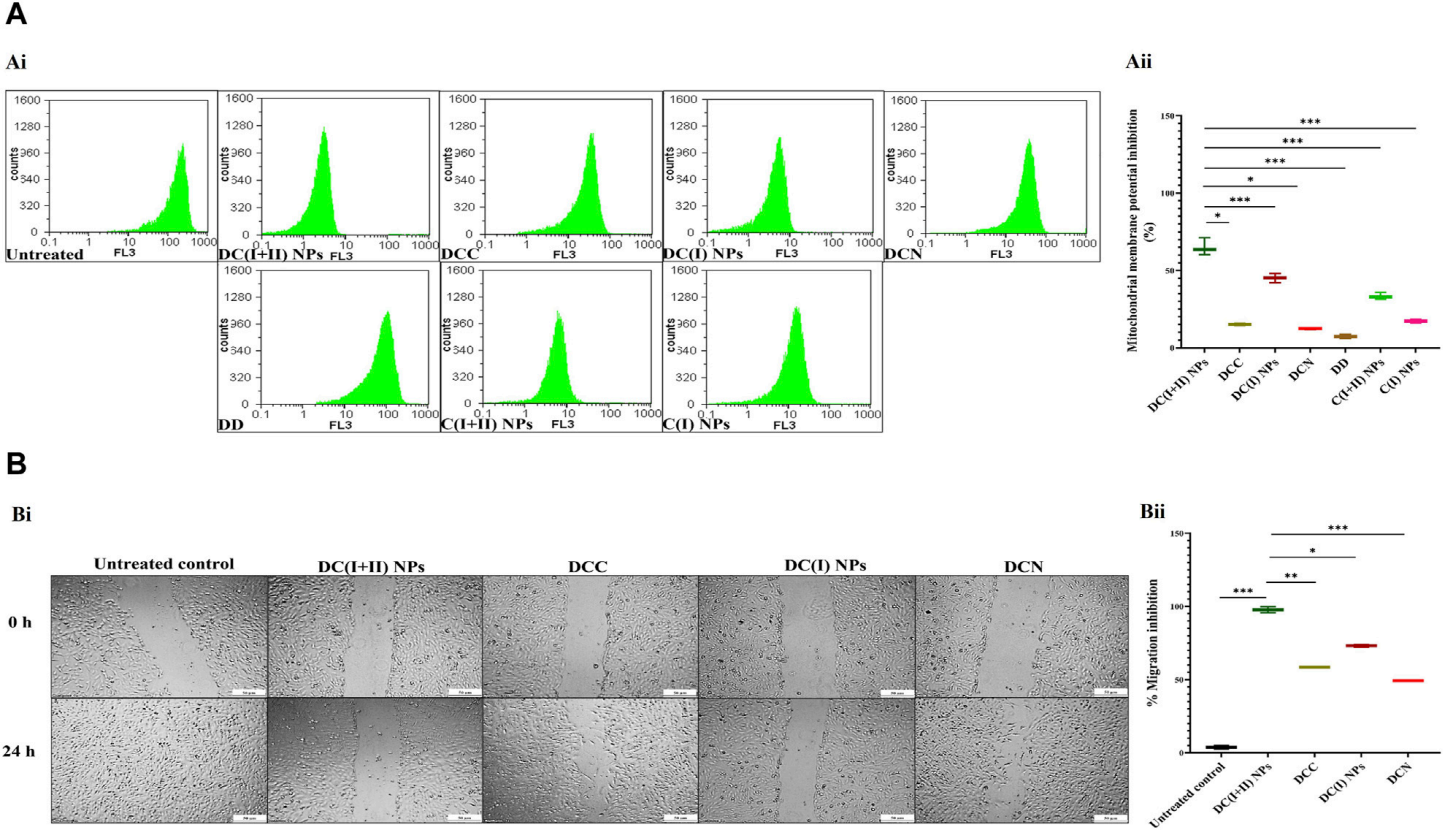
Mitochondrial damage and migration inhibition in the treated HepG2 cells. **(A)** Flow cytometry analysis of mitochondrial membrane potential (MP) damage in the treated HepG2 cells using tetramethylrhodamine (TMRE) staining, as illustrated by (Ai) flow charts and (Aii) the percentage of MP inhibition in terms of the relative percentages of low TMRE fluorescence intensity of the treated HepG2. **(B)** Anti-migration efficacy of nanocomplexes (nanocomplex of diethyldithiocarbamate (DD)-Cu_4_O_3_ nanoparticles (DC(I + II) NPs) and nanocomplex of DD-Cu_2_O NPs (DC(I) NPs)) compared to traditional DD-copper complexes (DCC and DCN) of DD with precursors of copper oxide NPs (copper chloride and copper nitrate, respectively), as demonstrated by (Bi) microscopic images of HepG2 cell migration (at 0 and 24 h) and (Bii) the percentage of migration inhibition. Data are shown as mean ± SD (n = 3). DC(I + II) NPs were compared to other tested compounds, and values are considered statistically significant at *p* < 0.05*, <0.005**, and <0.0001***. C(I + II) NPs and C(I) NPs; Cu oxides (Cu_4_O_3_ and Cu_2_O, respectively) NPs.

Regarding cuproptosis-mediated mitochondrial damage, nanocomplexes-treated HepG2 cells revealed the lowest MP (65.05% ± 3.26% and 45.24% ± 1.78%, respectively), followed by C(I + II) NPs (33.37% ± 1.33%), relative to other treated cells (Figures 3Ai, Aii). These results are evidenced by the lowest fluorescence intensity of TMRE with DC(I + II) NPs-treated HepG2 cells compared to those treated with DC(I) NPs (Figure 3Ai). Moreover, DC(I + II) NPs demonstrated the highest anti-migration potency on HepG2 cells (97.67% ± 2.03%), followed by DC(I) NPs (73.15% ± 0.89%), compared to the lowest percentages for their typical complexes (58.49% ± 0.28% and 49.21% ± 0.53%, respectively), as illustrated in Figures 3Bi, Bii.

### 3.3 Superior therapeutic potency of DC(I + II) NPs against metastatic HC (*in vivo* study) in terms of

#### 3.3.1 Morphology, weight, tumor markers, histology, immunostaining, and key gene expression of tumor tissues, as well as the proposed GSTP1 and MMP9 inhibition

**FIGURE 4:**
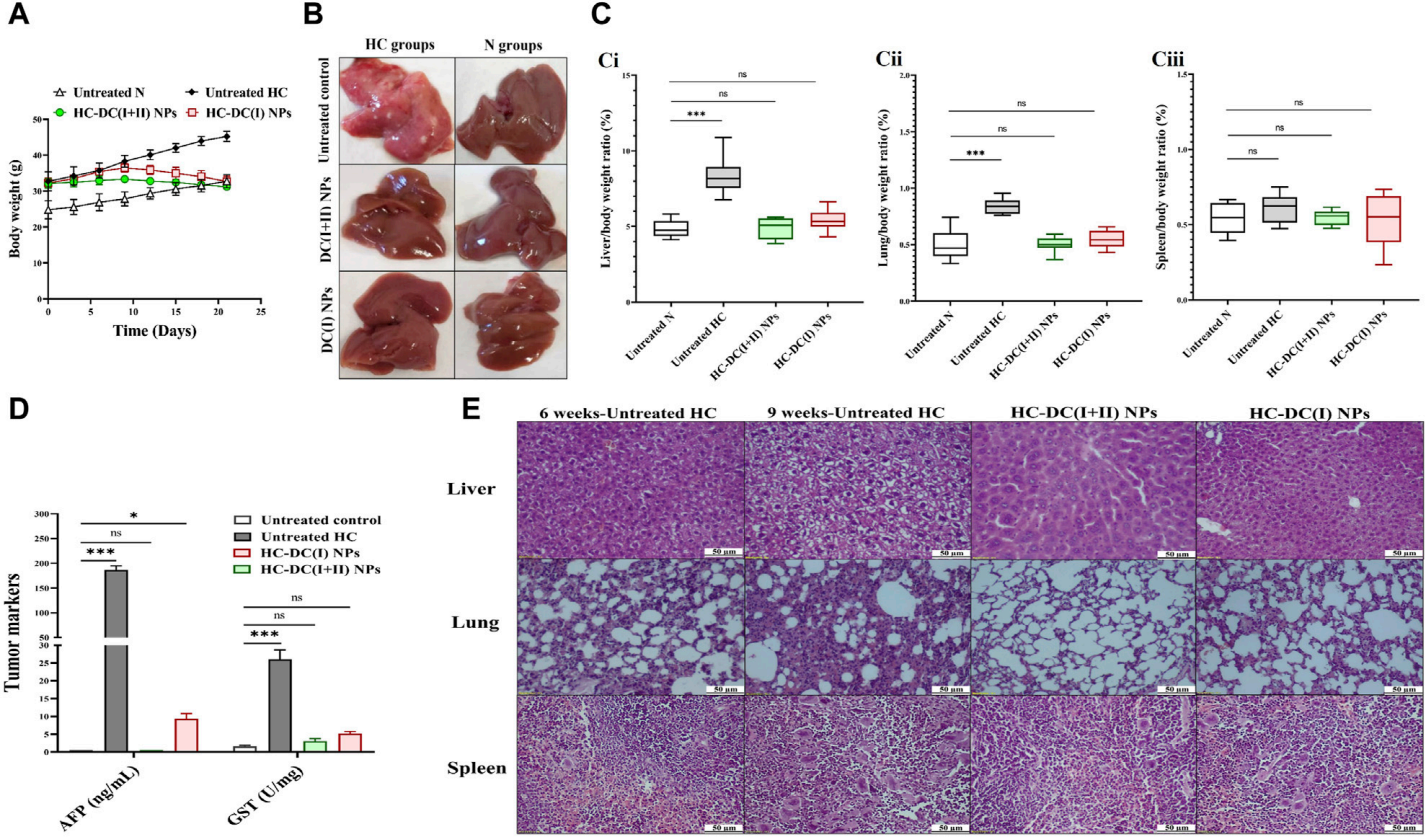
*In vivo* impact of nanocomplexes on the liver tumor in terms of weight, histological analysis, and tumor markers (α-fetoprotein (AFP) level and glutathione-S-transferase (GST) activity). **(A)** The measured body weight of mice during 3 weeks of treatment. **(B)** Liver morphology images of the untreated and treated hepatocellular carcinoma (HC) and normal (N) groups. **(C)** The relative weight of (Ci) liver, (Cii) lung, and **(B)** (Ciii) spleen to body weight of N, untreated HC, and nanocomplexes-treated HC [HC-diethyldithiocarbamate-Cu_4_O_3_ nanoparticles (DC(I + II) NPs) and HC-diethyldithiocarbamate-Cu_2_O nanoparticles (HC-DC(I) NPs)] groups. **(D)** Blood AFP level (ng/mL) and hepatic activity of GST (U/mg protein) in the untreated N, HC, and nanocomplexes-treated HC groups. **(E)** H&E-stained liver, lung, and spleen tissues of the untreated HC group at the sixth week of HC induction (showing neoplastic hepatocytes and a small area of lung cancer cells without alteration in the spleen) and at the ninth week of HC induction (demonstrating marked anaplasia in clear cell HC, a larger area of lung cancer, and nodular hyperplasia in spleen), and two nanocomplexes-treated HC groups after 6 weeks of induction and 3 weeks of treatment (showing a higher therapeutic impact of DC(I + II) NPs than DC(I) NPs for eradicating liver tumor cells and inhibiting metastasis to lung and spleen as well as normal spleen in both treated groups). Data are shown as mean ± SD (n ≥ 6). All the studied groups were compared to the untreated N group, and values are considered statistically significant at *p* < 0.05*, <0.005**, and <0.0001***.

**FIGURE 5:**
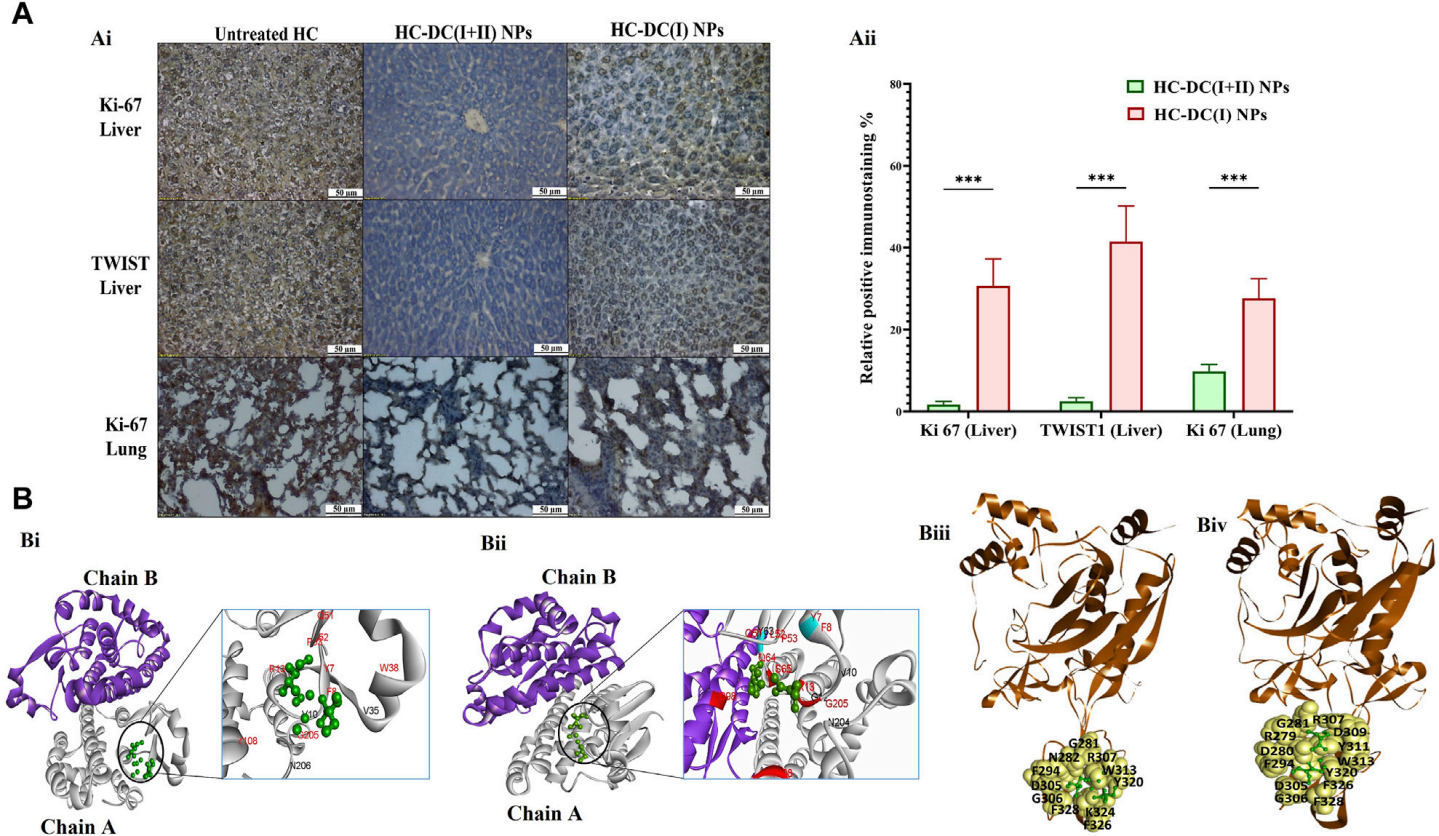
Immunohistological investigations for tumor markers in nanocomplexes-treated hepatocellular carcinoma mouse groups as well as computational inhibitory effect on GSTP1 and matrix metalloproteinase (MMP)9. **(A)** Ki-67 and TWIST1 immunostaining tumor tissues of the liver and lung in the untreated hepatocellular carcinoma (HC) group and nanocomplexes-treated HC [HC-diethyldithiocarbamate-Cu_4_O_3_ nanoparticles (DC(I + II) NPs) and HC-diethyldithiocarbamate-Cu_2_O nanoparticles (HC-DC(I) NPs)] groups, as demonstrated by (Ai) microscopic images and (Aii) the percentages of positive immunostained cells. Data are shown as mean ± SD (n ≥ 6). HC-DC(I + II) NPs group was compared to HC-DC(I) NPs groups, and values are considered statistically significant at *p* < 0.05*, <0.005**, and <0.0001***. **(B)** The computational results, as presented by (Bi, Bii) the docked complexes of GSTP1 (PDB ID: 3GUS, white “chain A” and purple “chain B”) with DC(I + II) or DC(I) (green-colored ball and stick style), respectively. The interface residues (black and red) in the docked complexes of GSTP1 with DC(I) and DC(I + II) are magnified and show the binding of the Cu complexes to the active site residues of GSTP1 (red), and (Biii, Biv) the docked complexes of MMP9 (PDB ID: 1L6J, brown-colored cartoon) with DC(I + II) or DC(I) (green-colored ball and stick style), respectively. The interface residues are represented by yellow-colored space-filling spheres labeled with black-colored amino acids. These docked complexes were given by the HDOCK server (http://hdock.phys.hust.edu.cn/) and visualized by Discovery Studio 2020 Client software.

Following induction of HC by DMAB and PB, oval nodules (the mean number∼21/liver organ) appeared only in the collected pale-colored livers of the untreated HC group. This group showed a 1.6-fold increase in body weight, ∼ 2-fold increase in weight of the liver and lung, no increment in spleen weight, as well as > 185-fold and >15-fold elevation in AFP level and GST activity, respectively, relative to the healthy N group (Figures 4A–D). Before starting treatment (at the sixth week), the induction of metastatic HC was investigated by H&E staining of liver, lung, and spleen tissues showing hepatic neoplastic changes (increased nuclear to cytoplasmic ratio and multinucleated giant cells), lung cancer cells, and no alterations in spleen (Figure 4E). At the end of the experiment (9 weeks), H&E staining of these tissues from the untreated HC-bearing animals demonstrated clear cell HC with marked anaplasia (irregular-shaped hyperchromatic nuclei and clear cytoplasm), an increasing area of lung cancer cells, and nodular lymphoid hyperplasia in the spleen (Figure 4E). Moreover, the results showed an elevation in the percentages of the Ki-67^+^-immunostaining and the metastatic marker (TWIST1^+^-immunostaining) in both liver and lung tissues (Figure 5Ai, Aii), indicating HC lung metastasis (Figure 5A).

The findings of the current study showed no differences in body weight, liver morphology or liver, lung, and spleen weights between the nanocomplexes-treated HC group and the untreated N group (Figures 4A–C). Both nanocomplexes (in the treated HC groups) also suppressed the AFP level (<10 ng/mL compared to >150 ng/mL in the HC untreated group); importantly, DC(I + II) NPs can normalize this tumor marker. Moreover, two nanocomplexes normalized the elevation in hepatic GST activity from 26.1 U/mg to <6 U/mg (Figure 4D). Histological and immunohistochemical investigations illustrated that DC(I + II) NPs had superior therapeutic efficacy against HC by showing normal hepatocytes with complete inhibiting metastasis to lung and normal splenic nodules, compared to DC(I) NPs (Figures 4E, 5A). As shown in Figures 5Ai, Aii, the treatment with this nanocomplex repressed the elevation of Ki-67 in the liver and lung as well as the metastatic marker (TWIST1) in the liver by 17.58, 2.810, and 16.56 folds, respectively, relative to DC(I) NPs. The latter showed significantly higher positive immunostaining percentages of Ki-67 and TWIST1 than DC(I + II) NPs (Figure 5A).

The inhibitory mechanism of the studied Cu complexes (DC(I + II) and DC(I)) on the activity of GSTP1 was studied using molecular docking analysis (Figures 5Bi,Bii). The retrieved 3D structure of GSTP1 is two chains: chain A and chain B, with 209 amino acid residues. The results showed that the two Cu complexes could bind to the enzyme with a superior binding affinity (Table 1) of DC(I + II), which bound firmly to the enzyme at different positions. The DC(I + II) interacted with 13 amino acid residues (9 of them are active site residues) of GSTP1 chain A. Whereas DC(I) is bound to 14 amino acid residues of chain A and one residue of chain B (11 of them are active site residues) of GSTP1 (Figures 5Bi, ii, respectively).

**TABLE 1:** The predicted binding affinity (solvation-free energy gain upon interface formation, Δ^i^G) between the studied enzymes and DC(I+II) or DC(I) in the obtained docked complexes.

Docked complex	Δ^i^G kcal/mol	Binding affinity between
MMP9_ DC(I+II)	−0.4	MMP9, DC(I+II)
MMP9_ DC(I)	−0.3	MMP9, DC(I)
DLAT_ DC(I+II)	−0.8	DLAT1, DC(I+II)
DLAT_ DC(I)	−0.2	DLAT1, DC(I)
DLAT1_DLAT2	−3.7	DLAT1, DLAT2
DLAT1_ DC(I+II)_DLAT2	−5.2	DLAT1, DLAT2
DLAT1_ DC(I)_DLAT2	−5.0	DLAT1_DLAT2
DLAT1_DLAT2_DLAT3	−0.1	DLAT2, DLAT3
DLAT1_ DC(I+II)_DLAT2_DLAT3	−3.7	DLAT2, DLAT3
DLAT1_ DC(I)_DLAT2_DLAT3	−2.5	DLAT2, DLAT3
SDH_DC(I+II)	−0.8	SDH, DC(I+II)
SDH_DC(I)	−0.6	SDH, DC(I)
SDH_(Fe-S)	−57	SDH, (Fe-S)
SDH_DC(I+II)_(Fe-S)	−56.8	SDH, (Fe-S)
SDH_ DC(I)_(Fe-S)	−54.1	SDH, (Fe-S)
ALDH2_DC(I+II)	−0.2	ALDH2, DC(I+II)
ALDH2_DC(I)	−0.0	ALDH2, DC(I)

The Δ^i^G was generated by the PDBePISA server, which is available at https://www.ebi.ac.uk/pdbe/pisa/; ALDH2 aldehyde dehydrogenase 2, DLAT dihydrolipoamide S-acetyltransferase, DC(I+II) diethyldithiocarbamate-Cu4O3 complex; DC(I) diethyldithiocarbamate-Cu2O complex, MMP9 matrix metalloproteinase 9, MTA1 metastasis-associated protein1, SDH succinate dehydrogenase. The Cu complex with the highest binding affinity to the tested enzymes are highlighted in light green.

MMP9 (gelatinase B) was also examined using *in silico* analysis, as shown in Figures 5Biii, Biv. The PDB 3D structure of MMP9 contains one chain with 425 amino acids. Both DC(I + II) and DC(I) complexes could bind to MMP9 with 13 and 11 amino acid residues, respectively (Figures 5Biii,Biv), and slightly higher affinity (Δ^i^G) to DC(I + II) (Table 1). However, neither DC(I + II) nor DC(I) could bind to the active site residues of the enzyme.

**FIGURE 6:**
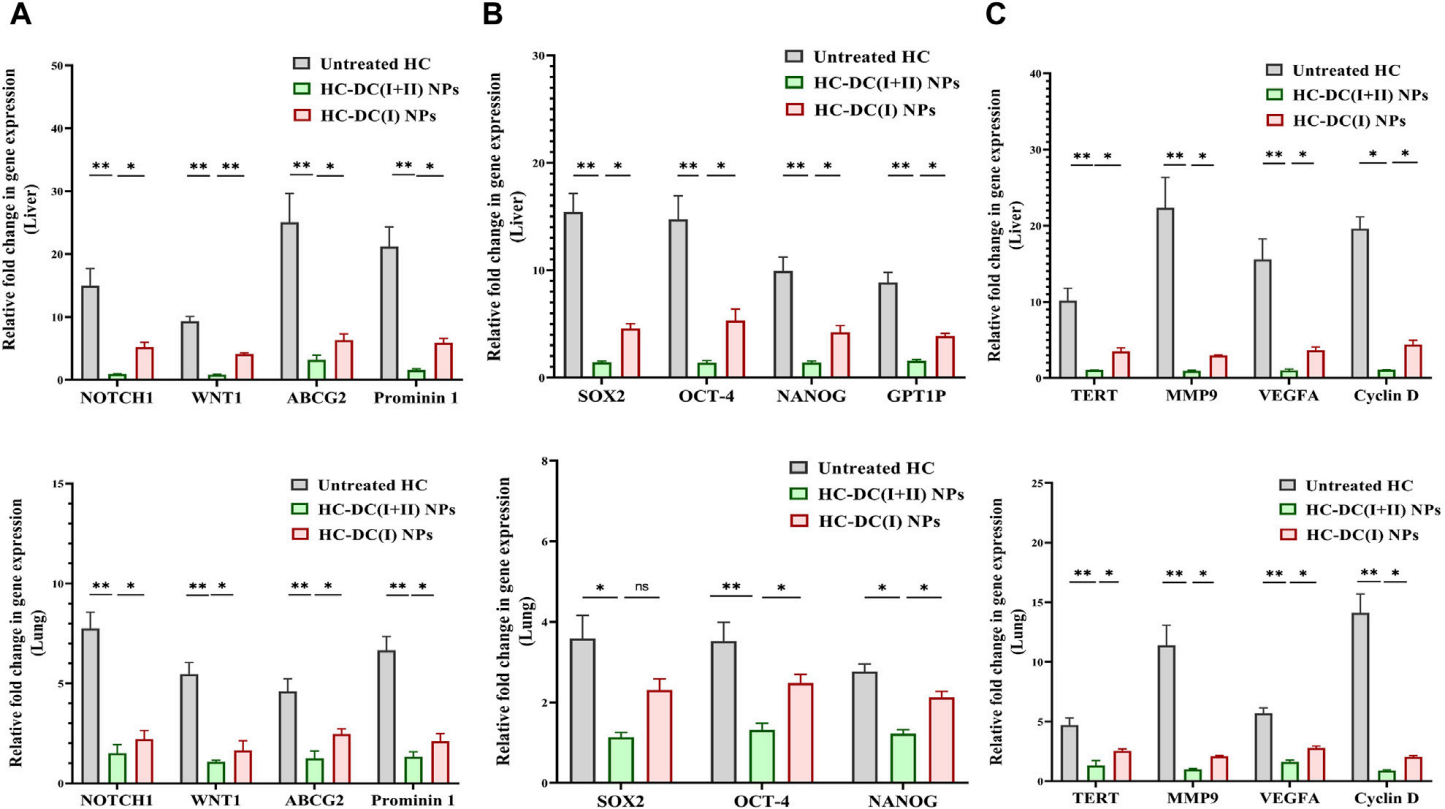
The inhibitory impact of nanocomplexes on fundamental stemness genes and oncogenes. Relative fold changes in gene expression of **(A)** NOTCH1, WNT1, ATP binding cassette subfamily G member 2 (ABCG2), and prominin 1 (CD133), **(B)** SOX2, OCT-4, NANOG, and glutathione-S-transferase (GST)P1, and **(C)** telomerase reverse transcriptase (TERT), matrix metalloproteinase (MMP)9, vascular endothelial growth factor (VEGF)A, and cyclin D in tumor tissues of the liver and lung in all the studied groups. The nanocomplex of diethyldithiocarbamate- Cu_4_O_3_ nanoparticles-treated hepatocellular carcinoma animal group (HC-DC(I + II) NPs) was compared to the untreated HC group and the nanocomplex of diethyldithiocarbamate-Cu_2_O nanoparticles-treated HC animal group (HC-DC(I) NPs). Data are shown as mean ± SD (n ≥ 6). Values are considered statistically significant at *p* < 0.05*, <0.005**, and <0.0001***.

Importantly, DC(I + II) NPs surpassed DC(I) NPs in downregulating key oncogene expression. DC(I + II) NPs repressed stemness genes (NOTCH1, WNT1, chemoresistance gene, prominin1, SOX2, OCT-4, and NANOG), GSTP1, telomerase, MMP9-stimulated metastasis, VEGFA, and cyclin D-mediated cell cycle by two to six folds, 5 folds, 2 folds, two to three folds, two to three folds, and two to four folds, respectively, in both tumor tissues (Figures 6A–C).

#### 3.3.2 Selective accumulation in tumor tissues, safety in normal tissues, and selective inhibition of mitochondrial lipoylated and Fe-S cluster enzymes with *in silico* analysis

**FIGURE 7:**
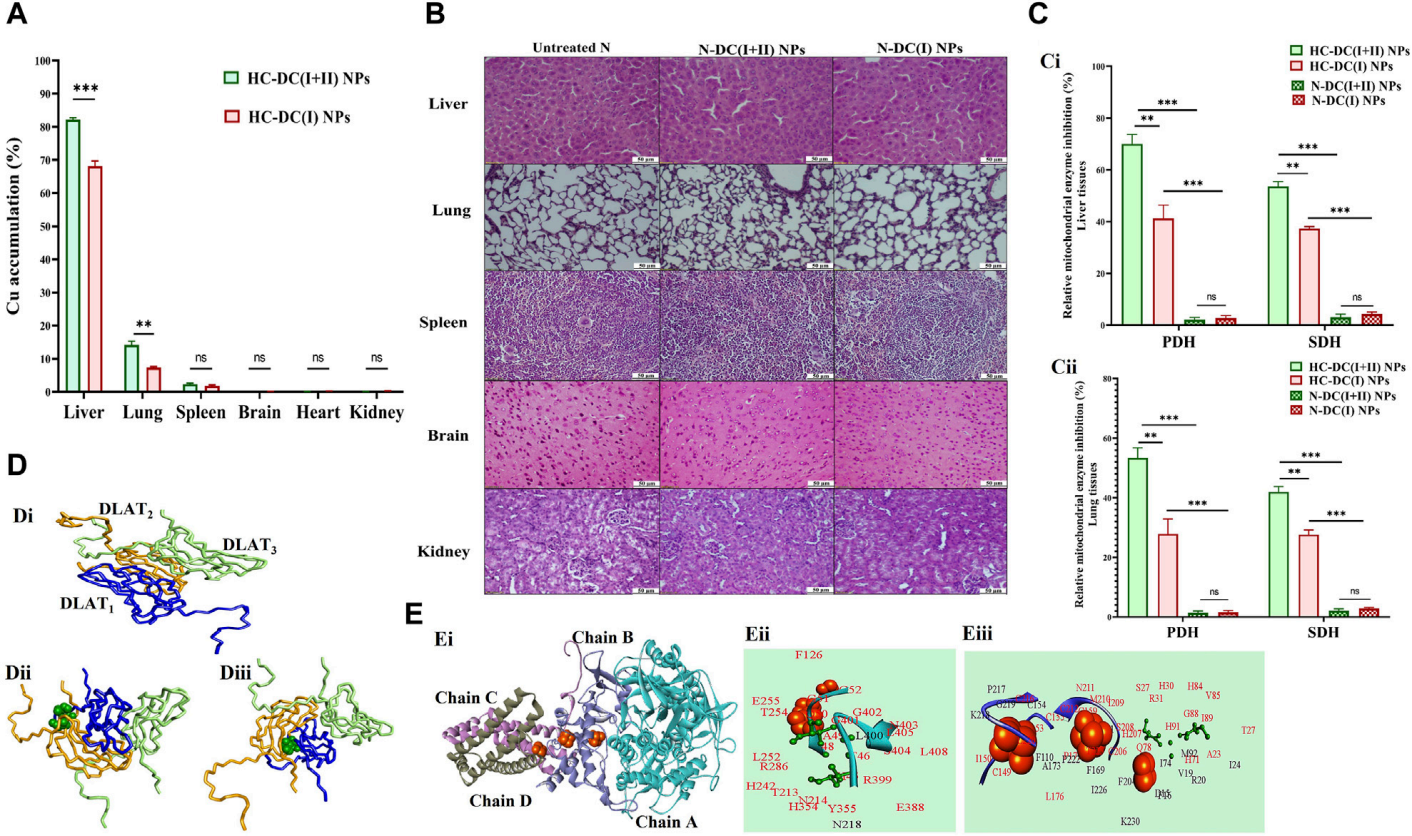
Selective accumulation in tumor tissues with a histological indication of safety in normal tissues and the cuproptosis-mediated inhibition of mitochondrial enzymes by the studied nanocomplexes with molecular docking analysis. **(A)** Atomic absorption spectroscopy results of nanocomplexes’ distribution in the liver, lung, spleen, brain, heart, and kidney after nanocomplexes’ injections into hepatocellular carcinoma (HC)-bearing mice. The nanocomplex of diethyldithiocarbamate-Cu_4_O_3_ nanoparticles-treated HC animal group (HC-DC(I + II) NPs) was compared to the nanocomplex of diethyldithiocarbamate-Cu_2_O nanoparticles-treated HC animal group (HC-DC(I) NPs). Data are shown as mean ± SD (n ≥ 6) and values were considered statistically significant at *p* < 0.05*, <0.005**, and <0.0001***. **(B)** H&E staining tissues of the untreated normal healthy mice (N) and treated N groups. **(C)** The relative inhibition percentage of pyruvate dehydrogenase (PDH) and succinate dehydrogenase (SDH) in nanocomplexes-treated HC groups and nanocomplexes-treated N groups in (Ci) liver and (Cii) lung tissues, respectively. Data are shown as mean ± SD (n ≥ 6). HC-DC(I + II) NPs group was compared to HC-DC(I) NPs and N-DC(I + II) NPs. Also, the HC-DC(I) NPs group was compared to the N-DC(I) NPs group, and the two N-nanocomplex groups were compared. The values are considered statistically significant at *p* < 0.05*, <0.005**, and <0.0001***. **(D)** Molecular docking with dihydrolipoamide S-acetyltransferase (DLAT, a component of PDH), as illustrated by (Di) the 3D structure of the docked complex of three DLAT (PDB: 1FYC, blue, orange, and light green wire style) molecules. (Dii, Diii) Docking models of DLAT_1_ (blue-colored wire style) with DC(I + II) or DC(I) (stick style and green-colored ball), respectively, followed by DLAT_2_ (orange-colored wire style), and then DLAT_3_ (light green-colored wire style). **(E)** Molecular docking with SDH as demonstrated by (Ei) the 3D structure of SDH (PDB: 1NEN, turquoise “chain A”, light blue “chain B”, violet “chain C”, and gray “chain D”-colored cartoons). (Eii, Eiii) The interacting residues (black and red) in the docked complexes of SDH with DC(I + II) and DC(I) (stick style and green-colored ball), respectively, are magnified and show the binding of the Cu complexes to the active site residues of SDH (red). The brown-colored space-filling spheres are referred to as the Fe-S centers of the SDH. The docked complexes were given by the HDOCK server (http://hdock.phys.hust.edu.cn/) and visualized by Discovery Studio 2020 Client software.

The nanocomplex biodistribution results illustrated that the investigated NPs were mostly accumulated in tumor tissues of the liver (>68%), followed by the lung (>14%), and then the spleen (≥2%), while other normal (non-tumor) tissues contained less than 0.09% (Figure 7A). Tumor uptake of DC(I + II) NPs was significantly higher in liver (82.20% ± 0.30%) and lung (14.17% ± 0.67%) tissues than that of DC(I) NPs (68.17% ± 0.88% and 7.32% ± 0.183%, respectively). This high tumor selectivity of nanocomplexes with their lowest uptake by normal tissues indicates the safety of these nanoformulations in normal tissues. These results were supported by the histological findings of the liver, lung, spleen, brain, and kidney tissues of normal mice treated with these nanocomplexes and revealed no alterations compared to the healthy N group (Figure 7B). Nanocomplexes of DC(I + II) and DC(I) selectively suppressed PDH (lipoylated enzyme) and SDH (Fe-S cluster protein) activities by > 41% and >27%, respectively, in both tumor tissues of the liver and lung, without affecting their activities in the corresponding tissues of the treated N groups (Figure 7C). The DC(I + II) nanocomplex inhibited hepatic PDH and hepatic SDH more effectively (38.10 ± 1.58 and 5.24 ± 0.07 U/mg protein, respectively) than the DC(I) nanocomplex (74.56 ± 2.18 and 7.07 ± 0.03 U/mg protein, respectively), compared to the untreated HC (126.89 ± 1.46 and 11.27 ± 0.45 U/mg protein, respectively). Also, in lung tissues, DC(I + II) NPs-treated HC animals had lower activities of these enzymes (40.20 ± 0.955 and 5.18 ± 0.05 U/mg protein, respectively) than the DC(I) NPs-treated HC mice (62.25 ± 1.46 and 6.46 ± 0.05 U/mg protein, respectively), relative to the untreated HC (86.26 ± 1.28 and 8.93 ± 0.25 U/mg protein, respectively).

The impact of both DC(I + II) and DC(I) on DLAT aggregation was evaluated using molecular docking analysis. DLAT is a single-chain 3D structure with 106 amino acid residues; it could bind to DC(I + II) with more affinity than DC(I) (Table 1). Binding of DLAT with these Cu complexes effectively increased the binding affinity between DLAT molecules (enhancing aggregation) with higher efficiency to DC(I + II) (Table 1). Analysis of the obtained docked complexes revealed that DC(I) could bind to 10 amino acid residues of DLAT_1_ and 17 amino acids of DLAT_2_, while DC(I + II) bound to DLAT_1_ with 7 amino acid residues and DLAT_2_ with 15 amino acid residues (Figure 7D).

The *in silico* analysis was also utilized to evaluate the probable iron displacement influence of DC(I + II) and DC(I) on SDH. The PDB-retrieved 3D structure of SDH has four subunits: A (succinate dehydrogenase flavoprotein, 588 amino acids), B (succinate dehydrogenase iron-sulfur protein, 238 amino acids), C (succinate dehydrogenase cytochrome b-556, 129 amino acids), and D (succinate dehydrogenase hydrophobic membrane anchor protein, 115 amino acids). The structure contains two dimeric (Fe_2_S_2_) and one tetrameric (Fe_4_S_4_) iron-sulfur centers that bind to chain B. The results showed that both the tested Cu complexes could bind to SDH with superior affinity to DC(I + II) (Table 1). DC(I) is bound to about 50 amino acid residues (of which 28 Fe-S binding residues) of the enzyme at chains B, C, and D. While DC(I + II) is bound to nearly 27 amino acid residues of the enzyme at chain A only. The binding affinity of the Fe-S to the enzyme was decreased from–57 kcal/mol to–56.8 kcal/mol and–54.1 kcal/mol after binding of DC(I + II) and DC(I), respectively, to the enzyme (Table 1). After comparing the interface residues of the obtained docked complexes with the enzyme active site residues, the outcomes revealed the binding of DC(I) and DC(I + II) complexes to 30 and 25 residues, respectively (Figure 7E).

#### 3.3.3 Selective prooxidant potential in tumor tissues and docking results

**FIGURE 8:**
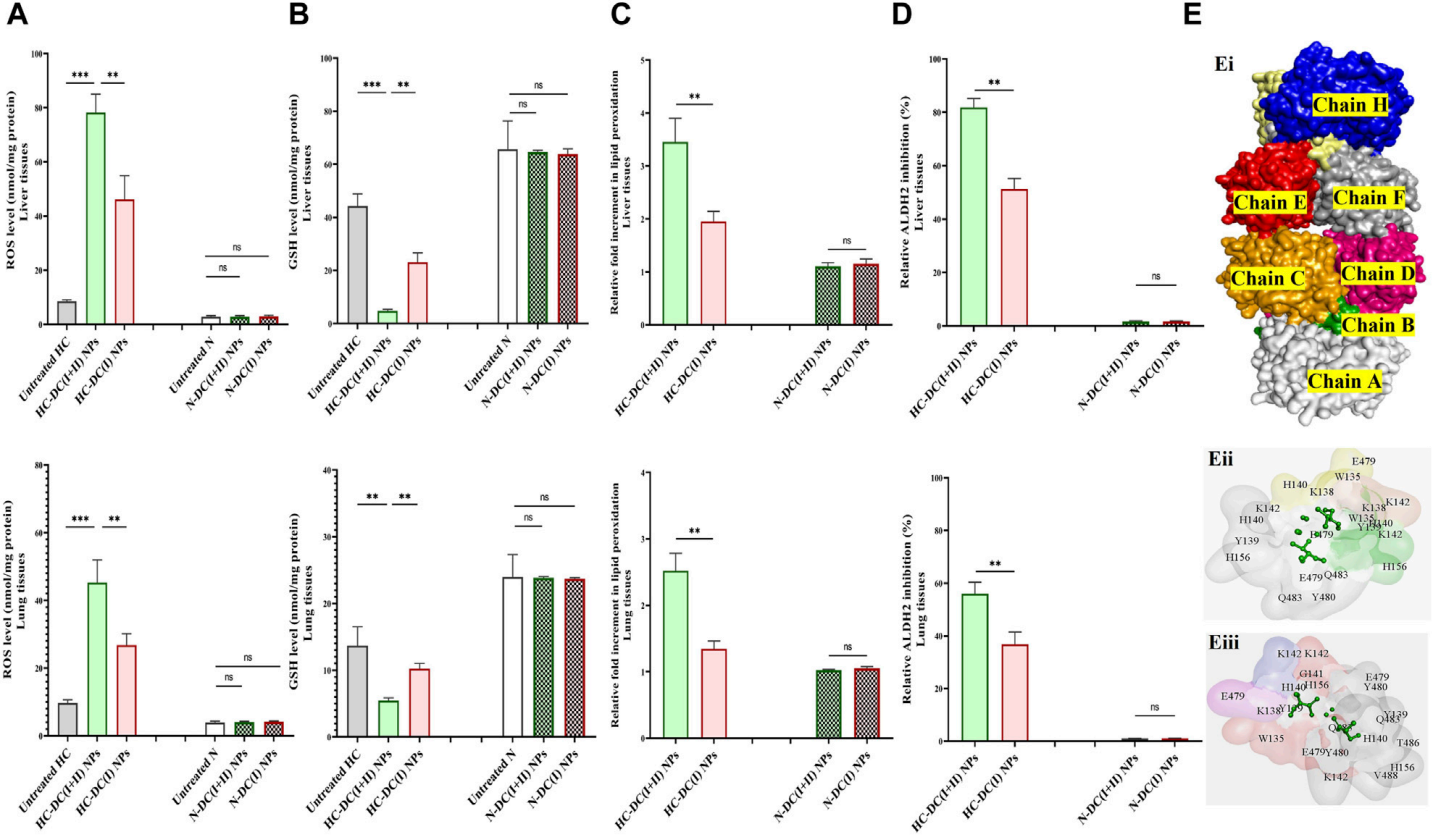
Selective oxidant impact of the studied nanocomplexes in the treated hepatocellular carcinoma (HC)-bearing mice as well as their molecular docking analysis with aldehyde dehydrogenase (ALDH) 2. **(A)** Reactive oxygen species (ROS) content (nmol/mg protein), **(B)** glutathione (GSH) level (nmol/mg protein), **(C)** relative fold increment of lipid peroxidation, and **(D)** relative ALDH2 inhibition in tumor tissues (liver and lung) of the untreated HC and treated HC groups (nanocomplex of diethyldithiocarbamate-Cu_4_O_3_ nanoparticles-treated HC (HC-DC(I + II) NPs) and nanocomplex of diethyldithiocarbamate-Cu_2_O nanoparticles-treated HC (HC-DC(I) NPs), as well as in normal corresponding tissues of the untreated and treated N groups. Data are shown as mean ± SD (n ≥ 6). The N group was compared to other groups, and the values are considered statistically significant at *p* < 0.05*, <0.005**, and <0.0001***. **(E)** Predicted inhibition of ALDH2, as shown by (Ei) the 3D structure of ALDH2 (PDB: 4FR8, white “chain A″, green “chain B″, orange “chain C″, pink “chain D″, yellow “chain E″, blue “chain F″, red “chain G″, and gray “chain H″-colored surface). (Eii, Eiii) Magnification of the interacting residues (gray) in the docked complexes of ALDH2 with diethyldithiocarbamate-Cu_4_O_3_ “DC(I + II)” or diethyldithiocarbamate-Cu_2_O “DCI)(I)” (stick style and green-colored ball), respectively.

Both nanocomplexes demonstrated selective elevation of ROS (≥5 folds) and lipid peroxidation (≥2.5 folds) with lowering GSH level (>1.3 fold) and inhibiting ALDH2 activity (>36%) in tumor tissues (liver and lung). These parameters showed no alterations in the respective normal tissues of mice in the nanocomplex-treated N groups (Figures 8A–D). The DC(I + II) NPs were more effective than DC(I) NPs in increasing ROS (9.1 and 4.7 folds) and lipid peroxidation (3.5 and 2.5 folds), as well as depleting GSH (9.3 and 2.51 folds) and ALDH2 activity (81.82% and 56.07%) in tumor tissues of the liver and lung, respectively.

The proposed inhibitory mechanisms of DC(I + II) and DC(I) on the activity of ALDH2 were determined by docking analysis. ALDH2 has eight chains (Figure 8Ei) with 500 amino acid residues in its 3D structure. The results revealed that both the investigated Cu complexes could bind to the enzyme at different chains and amino acids. Hence, DC(I) is bound to the enzyme with 21 amino acids at chains E, F, G, and H (Figure 8Eii), whereas DC(I + II) is attached to chains A, B, C, and D with 20 amino acids (Figure 8Eiii). The DC(I + II) complex interacted with the enzyme with a higher binding affinity (Table 1) than DC(I), but neither complex could bind to the enzyme active site residues.

#### 3.3.4 Normalization of liver function and hematological parameters

In comparison to the healthy N group, the liver tissue of animals in the untreated HC group showed a significant depletion in ALT and AST activities (2 and 1.28 folds, respectively) and albumin (1.31 folds). All of the aforementioned hepatic functional indices were normalized after the treatment with the investigated nanocomplexes (DC(I + II) NPs- and D(I) NPs-treated HC groups, Table 2. Furthermore, DC(I + II) NPs and D(I) NPs can ameliorate the HC-induced suppression in lymphocyte (lymph)% and elevation in monocyte (Mid)% and neutrophil (Gran)%. Moreover, no hematological variations were observed between nanocomplexes-treated N groups and healthy N group in all assessed parameters of RBCs, WBCs, and platelets (Table 2).

**TABLE 2:** Liver function and hematological parameters.

Liver function parameters								
Groups		ALT (U/mg)			AST (U/mg)		Albumin (g/dL)	
N		64.1±0.83			24.4±0.63		1.78±0.03	
Untreated HC		32.6±0.71*			19.1±1.12*		1.36±0.04*	
HC-DC(I+II) NPs		65.4±2.02			25.8±0.53		1.76±0.04	
HC-DC(I) NPs		71.2±2.99			27.9±0.58		1.77±0.04	
RBCs								
	RBC (10^6^/μL)	Hg (g/dL)	HCT (%)	MCV (fL)	MCH (pg)	MCHC (g/dL)	RDW-CV (%)	RDW-SD (fL)
N	7.25±0.09	12.2±0.05	35.8±0.85	50.4±0.92	17.0±0.02	34.3±1.15	15.4±1.25	27.9±1.62
N-DC(I+II) NPs	8.16±0.14	13.1±0.3	39.6±0.95	51.0±2.05	16.0±0.05	31.5±1.40	17.6±1.3	32.4±1.05
N-DC(I) NPs	7.51±0.11	12.5±0.25	39.0±3.00	57.0±1.95	16.6±0.15	29.2±1.25	15.2±1.25	27.2±0.65
Untreated HC	7.75±0.01	11.6±0.25	34.0±3.05	43.9±4.05	15.7±0.30	35.9±2.55	15.3±0.5	24.4±3
HC-DC(I+II) NPs	8.06±0.14	12.3±0.1	38.8±1.70	48.1±1.25	15.1±0.20	31.4±1.30	14.9±0.65	26.1±1.85
HC-DC(I) NPs	7.58±0.105	12.6±0.65	36.6±4.70	48.3±6.80	16.0±1.75	33.4±1.20	17.0±0.4	29.8±4.95
WBCs					PLT			
	WBC (10^3^/μL)	Lymph (%)	Mid (%)	Gran (%)	PLT (10^3^/μL)	MPV (fL)	PDW	PCT (mL/L)
N	9.16±0.20	83.8±0.08	5.58±0.67	10.6±0.59	1205±52	5.72±0.20	14.8±0.15	6.62±0.82
N-DC(I+II) NPs	9.80±0.59	86.6±0.86	5.06±0.71	8.31±1.57	1207±33	5.70±0.10	14.9±0.01	6.92±0.29
N-DC(I) NPs	8.61±0.21	87.1±1.01	4.22±0.65	8.69±0.36	1281±13	6.03±0.23	15.2±0.35	7.76±0.23
Untreated H	8.81±1.01	68.2±1.04*	10.9±0.55*	19.3±0.08*	1172±58	5.40±0.01	14.9±0.2	6.58±1.11
HC-DC(I+II) NPs	8.93±0.10	78.9±0.75	5.43±0.10	15.7±0.86	1197±3.0	5.55±0.15	15.4±0.05	7.06±0.04
HC-DC(I) NPs	9.90±0.43	84.3±0.84	3.89±0.02	11.8±0.82	1192±11	6.00±0.20	15.0±0.25	7.20±0.09

All values are expressed as mean±SD. All groups were compared to the normal healthy (N) mouse group and considered significantly different at p <0.05*, <0.005**, and <0.0001***. Liver function indexes were measured in liver homogenates, including ALT; alanine aminotransferase, and AST; aspartate aminotransferase. Hg; hemoglobin, HCT; hematocrit, MCV; mean corpuscular volume, fL; 10^-15^ liter, MCH; mean corpuscular Hg, MCHC; mean corpuscular Hg concentration, RDW-SD and RDW-CV; RBC distribution width-coefficient of standard deviation and variation, WBCs; white blood cells, Lymph; lymphocyte, Gran; granulocyte, Mid; monocytes, basophils, and eosinophils, MPV; mean PLT volume, PCT; plateletcrit, and PDW; PLT distribution width. Untreated HC; hepatocellular carcinoma-bearing mice, HC-DC(I+II) NPs; nanocomplex of diethyldithiocarbamate-Cu_3_O_4_ nanoparticles-treated HC animal group, HC-DC(I) NPs; nanocomplex of diethyldithiocarbamate-Cu_2_O nanoparticles-treated HC animal group; and the treated N groups (N-DC(I+II) NPs and N-DC(I) NPs).

## 4 Discussion

Because of mitochondrial importance as an energy provider (through TCA and the respiratory chain) for maintaining cancer stemness, cuproptosis (a novel regulated cell death manner dependent on mitochondrial stress) is deemed an effective therapeutic approach for halting CSC metastasis and self-renewal (Yadav et al., 2020). Cuproptosis thus exclusively targets apoptotic-resistant CSCs while having little effect on non-CSCs that rely mainly on anaerobic glycolysis to sustain their rapid proliferation (Loureiro et al., 2017; Yadav et al., 2020). Therefore, the Cu ionophore must have a suppressive effect on non-CSCs, such as pro-oxidant activity, to aggravate the cuproptotic effect. Two recent green chemically synthesized Cu oxide NPs (C(I + II) NPs and C(I) NPs) were chelated by DD, forming nanocomplexes of DC(I + II) NPs and DC(I) NPs. These semi-spherical-shaped DC(I + II) NPs and global-shaped DC(I) NPs whose elemental compositions were identified, demonstrated appropriate stability profiles in serum condition at 37°C (Figures 1A–D) as well as higher safety on normal liver cells than their corresponding complexes and DD. Recently, these nanocomplexes exhibited high anti-metastatic effects with suppressing ALDH1A activity and elevating ROS levels in cancer cells (Abu-Serie and Eltarahony, 2021; Abu-Serie and Abdelfattah, 2023). In line with these recent findings, the current study revealed their highest growth inhibitory potential against both liver cancer cell lines, compared to C(I + II) NPs, C(I) NPs, DD, and their corresponding typical complexes, as well as their most potent anti-migratory efficacy. More importantly, both nanocomplexes showed the highest cuproptotic potential in the treated HepG2 cells, as evidenced by the lowest MP (65.05% and 45.24%, respectively, Figure 3A). This could be attributed to the nanocomplex nanosizes that positively affect cellular uptake of their included Cu, as well as the GSH-suppressive effect of DD, where GSH depletion increases cancer cells’ sensitivity to cuproptosis (Abu-Serie and Eltarahony, 2021; Li et al., 2022; Tsvetkov et al., 2022; Abu-Serie and Abdelfattah, 2023). Interestingly, this study is the first to evaluate the cuproptotic efficacy of these promising nanocomplexes against metastatic HC.

The DC(I + II) exhibited significantly higher therapeutic potential than DC(I) not only toward cells but also against the metastatic HC animal model (in terms of histological investigations and tumor markers “AFP, GST, Ki-67, CSC genes, and metastasis mediators “TWIST1-induced epithelial-mesenchymal transition (EMT), and MMP9”). Due to their nanosizes, both nanocomplexes had a higher accumulation rate in tumor tissues than normal tissues, but DC(I + II) NPs showed greater distribution percentages in tumor tissues than DC(I) NPs (Figure 7A). This may be owing to DC(I + II) NPs have a lower negative surface charge than DC(I) NPs, which results in superior binding to the negatively charged tumor cell plasma membrane (Frohlich, 2012). In the HC-DC(I + II) NPs group, the highly accumulated Cu in only tumor tissues, as the main initiator of selective cuproptosis, led to more aggregating lipoylated enzymes (e.g., PDH) and more destabilization of Fe-S cluster protein (e.g., SDH), resulting in high mitochondrial stress. Interestingly, docking results revealed that the DC(I + II) complex had a higher affinity for DLAT of PDH and enhanced its aggregation more than the DC(I) complex. Furthermore, both complexes could inhibit competitively the activity of this enzyme and decrease its affinity for Fe-S. The computational findings also showed that DC(I) had more capability to reduce the affinity between SDH and Fe-S (Fe displacement) than DC(I + II). However, DC(I + II) showed a higher affinity for SDH (Table 1) and suppressed SDH activity more effectively than DC(I) (Figures 7Ci, Cii). In addition to higher Cu content in HC-treated HC-DC(I + II) NPs’ tumor tissues, the freed Fe from Cu displacement in the Fe-S cluster (Table 1) can induce the Fenton reaction (Vallieres et al., 2017), causing oxidative stress. Liver tumor cells defend against the generated oxidative stress products by elevating the expression of ALDH2 (Abu-Serie, 2023) and GST, practically GSTP1. The latter is closely coincident with malignant transformation of liver cells and mediates chemoresistance via conjugating proapoptotic drugs with GSH (Muramatsu and Sakai, 2006; Sell, 2008; Li et al., 2013; Mazari et al., 2023). Furthermore, AFP which promotes HC’s malignant transformation, involves in the process of multidrug resistance and acts as an immunosuppressor (Li et al., 2021). Therefore, suppression of these tumor markers represents potential therapeutic targets. Thiol affinity of DD may be attributed to the inhibition potency of both complexes for ALDH2 and GST activities by forming disulfide adducts with cysteine residues of catalytically active sites of these enzymes. Additionally, a recent study illustrated that DD can inhibit transcriptional activation of nuclear factor erythroid-2, which induces GSTP1 expression (Satoh et al., 2002; Abu-Serie and Abdelfattah, 2022).

Telomerase reverse transcriptase is also essential for maintaining cancer stemness and stimulates the conversion of non-CSCs to CSCs by protecting them from oxidative stress and inducing VEGF expression-mediated angiogenesis and EMT-mediated metastasis via the promotion of NF-κB-dependent MMP expression (Kong et al., 2014; Zou et al., 2020). As demonstrated in the current study, DC(I + II) NPs inhibited not only the expression of stemness genes but also the TERT gene and its downstream genes (MMP9 and VEGF2) in both tumor tissues (Figures 6A–C). The *in silico* results revealed a higher binding affinity of DC(I + II) to MMP9 than DC(I), as well as the ability of these nanocomplexes to inhibit its activity via non- or uncompetitive mechanisms due to their proposed inability to bind the enzyme active sites (Figures 5Biii, Biv).

Besides the thiol affinity-dependent oxidant activity of DD resulting in GSH inactivation (Skrott and Cvek, 2012), Cu(II) of Cu_4_O_3_ “(Cu^+1^)_2_ (Cu^+2^)_2_ O_3_” in DC(I + II) NPs depleted the GSH level by catalyzing its oxidation, producing its oxide form (GSSG) and Cu (I) (Ngamchuea et al., 2016). Consequently, tumor tissues of HC-DC(I + II) NPs had a lower level of GSH than those of DC(I) NPs (Figure 8B). The released Cu(I) or Cu (I) of DC(I) NPs could directly generate hydroxyl radicals via the Fenton reaction, but DC(I + II) NPs-treated tumor tissues had a lesser antioxidant defense, resulting in more oxidative damage (lipid peroxidation) than DC(I) NPs. Moreover, DC(I + II) NPs revealed stronger inhibition potency on mitochondrial ALDH2 activity than DC(I) NPs (Figures 8D, E; Table 1). The predicted analysis also supported that DC(I + II) had a higher binding affinity to ALDH2 than DC(I), and the mechanism of inhibition by both nanocomplexes was non- or uncompetitive since they did not bind to the enzyme active site residues (Figures 8Eii, Eiii). In addition to being a CSC marker, ALDH2 is crucial functional regulator that enhances hepatic CSC self-renewal, proliferation, and metastasis by increasing the expression of SOX2, OCT-4, and NANOG and activating EMT (Chen et al., 2020; Zhang and Fu, 2021). It also acts a CSC protector by detoxifying reactive aldehydes, such as those generated by lipid peroxidation (Zhang and Fu, 2021). Therefore, its inhibition causes stemness suppression and elevates lipid peroxidation. Accordingly, following treatment with DC(I + II) NPs as opposed to its counterpart, stemness gene expressions were more repressed and cellular lipid peroxidation content was higher. In clinical trials (phase I/II), disulfiram (parent compound of DD) demonstrated a safe and effective response in increasing cancer patients’ survival rates (Kang et al., 2023). Elesclomol (cuproptosis inducer) alone or in combination with paclitaxel had a favourable safety profile but a low response rate (Zheng et al., 2022).

## 5 Conclusion

In comparison with DC(I) NPs, DC(I + II) nanocomplex demonstrated a significantly higher accumulation rate in tumor tissues, inhibition-dependent cuproptotic activity for mitochondrial enzymes, and depletion of antioxidant indices (GSH and ALDH2). As a result of these effects, the DC(I + II) nanocomplex exhibited a more potent anti-metastatic HC impact, as evidenced by histological, immunohistological, molecular, and biochemical investigations for key tumor, stemness gene, and metastasis markers. Both nanocomplexes showed high selectivity against tumor tissues without causing any alterations in normal healthy cells. Consequently, DC(I + II) NPs could be promising against a variety of aggressive tumors.

## Data Availability

The original contributions presented in the study are included in the article/Supplementary Material, further inquiries can be directed to the corresponding author.
