# Supplementary material for: Superior cuproptotic efficacy of diethyldithiocarbamate-Cu4O3 nanoparticles over diethyldithiocarbamate-Cu2O nanoparticles in metastatic hepatocellular carcinoma
Source: Front Pharmacol. 2024 Jul 15;15:1388038. doi: 10.3389/fphar.2024.1388038 (PMC11284037; doi:10.3389/fphar.2024.1388038)
Supplement: Supplementary file 1 [file Table1.DOCX]

**Supplementary Table 1 Forward (F) and reverse (R) primers of the investigated genes**

| **Gene** | **Primers** |
| --- | --- |
| NOTCH1 | F: 5′-CCCTTGCTCTGCCTAACGC-3′  R: 5′-GGAGTCCTGGCATCGTTGG-3′ |
| WNT1 | F: 5′-GGTTTCTACTACGTTGCTACTGG-3′  R: 5′-GGAATCCGTCAACAGGTTCGT-3′ |
| ABCG2 | F: 5′-GAACTCCAGAGCCGTTAGGAC-3′  R: 5′-CAGAATAGCATTAAGGCCAGGTT-3′ |
| Prominin1 (CD133) | F: 5′-ACTGGGGCTGTGTGGAAAG-3′  R: 5′- GCATTGAAGGTATCTTGGGTCTC-3′ |
| SOX2 | F: 5′-CCGTGATGCCGACTAGAAAAC-3′  R: 5′-AGCGCCTAACGTACCACTAG-3′ |
| OCT-4 | F: 5′-CCCACTTCACCACACTCTACT-3′  R: 5′-GCTCCAGGTTCTCTTGTCTA-3′ |
| NANOG | F:5′-CCACCAGGTGAAATATGAGAC-3′  R: 5′-GGCTCACAACCATACGTAAC-3′ |
| GSTP1 | F:5′-TCTACGCAGCACTGAATCCG-3′  R: 5′-GCCCTCGAACTGGGAAGTAG-3′ |
| TERT | F: 5′-GCACTTTGGTTGCCCAATG-3′  R: 5′-GCACGTTTCTCTCGTTGCG-3′ |
| MMP9 | F: 5′-GCGTCGTGATCCCCACTTAC-3′  R: 5′-CAGGCCGAATAGGAGCGTC-3′ |
| VEGFA | F: 5′-GTGAGGTGTGTATAGATGTGGGG-3′  R: 5′-ACGTCTTGCTGAGGTAACCTG-3′ |
| Cyclin D | F:5′-CAGAAGTGCGAAGAGGAGGTC-3′  R: 5′-TCATCTTAGAGGCCACGAACAT-3′ |

ABCG2: ATP Binding Cassette Subfamily G Member 2, GSTP1: glutathione S-transferase pi 1, TERT: telomerase reverse transcriptase, MMP9: matrix metalloproteinase 9, and VEGFA: vascular endothelial growth factor.
